# Supplementary material for: An open-source framework for large-scale, flexible evaluation of biomedical text mining systems
Source: J Biomed Discov Collab. 2008 Jan 29;3:1. doi: 10.1186/1747-5333-3-1 (PMC2276192; doi:10.1186/1747-5333-3-1)
Supplement: Additional file 1 — Evaluation platform source code. The additional file contains the software evaluation framework discussed in this paper. The most current release can be downloaded from SourceForge.net [1]. See the accompanying README file for instructions on installation and use. Also included in the distribution is a collection of UIMA wrappers for some commonly used BioNLP tools and annotated corpora. [file 1747-5333-3-1-S1.zip › bionlp_uima_repository-1.0.1/docs/api/src-html/edu/uchsc/ccp/util/nlp/annotation/Span.html]

```
001    /*
002     *
003     * The Original Code is UCHSC Utilities.
004     *
005     * The Initial Developer of the Original Code is University of Colorado.  
006     * Copyright (C) 2005.  All Rights Reserved.
007     *
008     * UCHSC Utilities was developed by the Center for Computational Pharmacology
009     * (http://compbio.uchsc.edu) at the University of Colorado Health 
010     *  Sciences Center School of Medicine with support from the National 
011     *  Library of Medicine.  
012     *
013     *
014     * Contributor(s):
015     *   William A. Baumgartner <william.baumgartner@uchsc.edu> (Original Author)
016     *   
017     * Created: January 2006
018     * Dependencies:
019     * TODO:
020     * Changes:
021     *      
022     */
023    
024    package edu.uchsc.ccp.util.nlp.annotation;
025    
026    import edu.uchsc.ccp.util.nlp.annotation.comparison.SloppySpanComparator;
027    import edu.uchsc.ccp.util.nlp.annotation.comparison.SpanComparator;
028    import edu.uchsc.ccp.util.nlp.annotation.comparison.StrictSpanComparator;
029    
030    public class Span implements Comparable {
031    
032        private int spanStart;
033    
034        private int spanEnd;
035    
036        public Span(int spanStart, int spanEnd) throws InvalidSpanException {
037            this.spanStart = spanStart;
038            this.spanEnd = spanEnd;
039            validateSpan();
040        }
041    
042        public int getSpanEnd() {
043            return spanEnd;
044        }
045    
046        public void setSpanEnd(int spanEnd) throws InvalidSpanException {
047            this.spanEnd = spanEnd;
048            validateSpan();
049        }
050    
051        public int getSpanStart() {
052            return spanStart;
053        }
054    
055        public void setSpanStart(int spanStart) throws InvalidSpanException {
056            this.spanStart = spanStart;
057            validateSpan();
058        }
059    
060        private void validateSpan() throws InvalidSpanException {
061            if (spanStart > spanEnd) {
062                throw new InvalidSpanException("Invalid span: Start of span is greater than end of span. spanStart=" + spanStart + "  spanEnd="
063                        + spanEnd);
064            }
065        }
066    
067        /**
068         * Return the length of the span
069         * 
070         * @return
071         */
072        public int length() {
073            return spanEnd - spanStart;
074        }
075    
076        /**
077         * The default equals() method uses the StrictSpanComparator
078         */
079        @Override
080        public boolean equals(Object spanToEquate) {
081            if (!(spanToEquate instanceof Span)) {
082                throw new ClassCastException("A Span object expected.");
083            } else {
084                SpanComparator sc = new StrictSpanComparator();
085                Span span = (Span) spanToEquate;
086    
087                if (sc.compare(this, span) == 0) {
088                    return true;
089                } else {
090                    return false;
091                }
092            }
093        }
094    
095        /**
096         * The overlaps() method uses the SloppySpanComparator
097         * 
098         * @param overlappingSpan
099         * @return
100         */
101        public boolean overlaps(Span overlappingSpan) {
102            SpanComparator sc = new SloppySpanComparator();
103            if (sc.compare(this, overlappingSpan) == 0) {
104                return true;
105            } else {
106                return false;
107            }
108        }
109    
110        /**
111         * Returns true if this span starts before the inputted span, false otherwise. (Equal span starts result in false).
112         * 
113         * @param span
114         * @return
115         */
116        public boolean startsBefore(Span span) {
117            if (this.spanStart < span.getSpanStart()) {
118                return true;
119            } else {
120                return false;
121            }
122        }
123    
124        /**
125         * Returns true if this span ends before the inputted span, false otherwise. (Equal span ends result in false).
126         * 
127         * @param span
128         * @return
129         */
130        public boolean endsBefore(Span span) {
131            if (this.spanEnd < span.getSpanEnd()) {
132                return true;
133            } else {
134                return false;
135            }
136        }
137    
138        @Override
139        public int hashCode() {
140            return this.toString().hashCode();
141        }
142    
143        @Override
144        public String toString() {
145            return "[" + this.spanStart + ".." + this.spanEnd + "]";
146        }
147    
148        @Override
149        public Span clone() {
150            Span newSpan = null;
151            try {
152                newSpan = new Span(this.spanStart, this.spanEnd);
153            } catch (InvalidSpanException e) {
154                e.printStackTrace();
155            }
156            return newSpan;
157        }
158    
159        public int compareTo(Object spanToEquate) {
160            if (spanToEquate instanceof Span) {
161                SpanComparator sc = new StrictSpanComparator();
162                Span span = (Span) spanToEquate;
163    
164                return sc.compare(this, span);
165            } else {
166                System.err.println("Error, cannot compare a Span to a " + spanToEquate.getClass().getName());
167                return -1;
168            }
169        }
170    
171    }
```
